# High‐Resolution Electronic Excitation and Emission Spectra of Pentacene and 6,13‐Diazapentacene Monomers and Weakly Bound Dimers by Matrix‐Isolation Spectroscopy

**DOI:** 10.1002/chem.202003999

**Published:** 2020-12-22

**Authors:** Jean Thusek, Marvin Hoffmann, Olaf Hübner, Stefan Germer, Hendrik Hoffmann, Jan Freudenberg, Uwe H. F. Bunz, Andreas Dreuw, Hans‐Jörg Himmel

**Affiliations:** ^1^ Anorganisch-Chemisches Institut Ruprecht-Karls-Universität Heidelberg Im Neuenheimer Feld 275 69120 Heidelberg Germany; ^2^ Interdisziplinäres Zentrum für Wissenschaftliches Rechnen Ruprecht-Karls-Universität Heidelberg Im Neuenheimer Feld 205 69120 Heidelberg Germany; ^3^ Organisch-Chemisches Institut Ruprecht-Karls-Universität Heidelberg Im Neuenheimer Feld 270 69120 Heidelberg Germany

**Keywords:** dimerisation, electronic structure, heterocycles, matrix isolation, pentacene

## Abstract

N‐Heteropolycycles are among the most promising candidates for applications in organic devices. For this purpose, a profound understanding of the low‐energy electronic absorbance and emission characteristics is of crucial importance. Herein, we report high‐resolution absorbance and fluorescence spectra of pentacene (**PEN**) and 6,13‐diazapentacene (**DAP**) in solid neon obtained using the matrix‐isolation technique. Accompanying DFT calculations allow the assignment of specific vibrationally resolved signals to corresponding modes. Furthermore, we present for the first time evidence for the formation of van der Waals dimers of both substances. These dimers exhibit significantly different optical characteristics resulting from the change of electronic properties evoked by the incorporation of sp^2^ nitrogen into the molecular backbone.

## Graphical abstract

**High‐resolution and fluorescence** spectra of pentacene and 6,13‐diazapentacene isolated in solid neon are presented. In alliance with quantum‐chemical calculations, the spectra give valuable information about the monomeric compounds. In addition, the matrix‐isolation technique was used to generate weakly bound dimers which were spectroscopically studied for the first time, providing information about the electronic coupling therein.

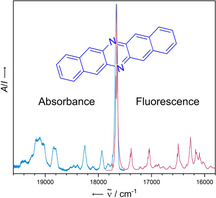

## Introduction

Acenes, polyaromatic hydrocarbons composed of linearly annulated benzene units,^[1]^ receive much interest from theoretical and experimental scientists.[^2^, ^3^, ^4^, ^5^] The smaller species of this class have been intensely studied.[^6^, ^7^, ^8^, ^9^, ^10^, ^11^, ^12^, ^13^, ^14^] Synthetic approaches, based on the photochemical bisdecarbonylation of bridging α‐diketones, the thermal deoxygenation of endoxide precursors or the thermal cleavage of covalent dimers, have made longer acenes accessible,[^15^, ^16^, ^17^, ^18^] up to the on‐surface generation of dodecacene.^[19]^

Particular focus has been devoted to the application of large polyaromatic hydrocarbons[^5^, ^20^, ^21^] and acenes, in particular[^22^, ^23^, ^24^, ^25^, ^26^] in organic electronics. Their performance in devices such as organic field‐effect transistors (OFETs), organic light‐emitting diodes (OLEDs) or organic photovoltaic devices (OPV) depends on solid‐state packing, reorganisation energy and intermolecular electronic coupling influencing the charge‐carrier mobility. Limiting factors are synthetic accessibility, solubility, photostability and oxidative resistance. The improved electronic characteristics of the longer acenes come at the price of reduced stability, impeding application. Pentacene still remains a promising and widely used material in the field of organic materials due to its relative stability combined with sufficient field‐effect mobility.^[27]^ It displays a reorganisation energy as low as 59 meV.^[28]^ Quasi‐monolayer semiconductors on poly(amic acid) surfaces gave charge carrier mobilities of 7.6 cm^2^ V^−1^ s^−1^ in the linear region.^[29]^

Substituted acenes gain importance in materials research. Two substitution strategies exist: Firstly, end‐on substitutions relative to the long axis of the acene tune the dipole characteristics with a push–pull substitution pattern.^[30]^ Alternatively, side‐chain substituents on the central ring (Anthony et al.^[22]^) improve stability towards oxidation and processability.[^31^, ^32^] Secondly, a (formal) substitution of one or more CH units by sp^2^ nitrogen atoms creates electron‐deficient systems compared to the unsubstituted acenes. Energetic position of frontier orbitals and band gaps are easily tunable.[^33^, ^34^] Combination of these two strategies creates stable, easily processable materials with desired electronic and crystallisation characteristics.[^35^, ^36^, ^37^, ^38^, ^39^]

**Scheme 1:**
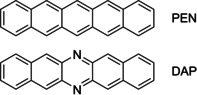
Lewis structures of pentacene (**PEN**) and 6,13‐diazapentacene (**DAP**) studied in the present work.

Herein, we provide optical spectra of pure pentacene (**PEN**) and of its nitrogen‐substituted analogue 6,13‐diazapentacene (**DAP**, Scheme 1) in solid neon using the matrix isolation technique.[^40^, ^41^, ^42^] This method allows the creation of diluted samples while minimising extrinsic effects such as solvation or aggregation. The substances trapped in solid noble gas matrices at low temperature (4 K) are analysed by standard spectroscopic methods (vis and fluorescence). At low concentrations, matrices serve as models for gas phase analyses that are not always feasible. Absorbance measurements on solid‐state matrices of noble gases have, in turn, the advantage of avoiding hot bands. Narrow band widths result simplifying interpretation. This technique is powerful in terms of the simulation of gas phase analytics and the deconvolution of the vibronic states in the absorbance spectra and was hitherto applied to a series of higher acenes, including pentacene, in solid argon by Bettinger et al.^[43]^ as well as tetracene and azatetracenes in neon.^[44]^ Here, we extend our studies to the pentacene analogues trapped in solid neon (Scheme 1).

The dependence of the absorbance characteristics of **PEN** from different matrix materials has previously been studied by Allamandola et al. alongside with its cationic form and the selective formation of the anion by using alkali metals as matrix dopants.^[45]^ In this work, we present for the first time fluorescence spectra of (matrix‐isolated) **PEN** monomers and assign all bands/signals in the electronic excitation and emission spectra with the help of quantum‐chemical calculations. Even more importantly, we obtained positive proof for the formation of weakly bound dimers and aggregates by series of experiments with variable concentrations and annealing cycles. Last but not least and probably most importantly, we do not stop with the analysis of **PEN**, but report for the first time a full matrix‐isolation analysis (electronic excitation and emission spectra) of **DAP** monomers, weakly bound dimers and aggregates, backed by the results of detailed quantum‐chemical calculations.

## Results and Discussion

### Comparison of UV/Vis methods

**Figure 1:**
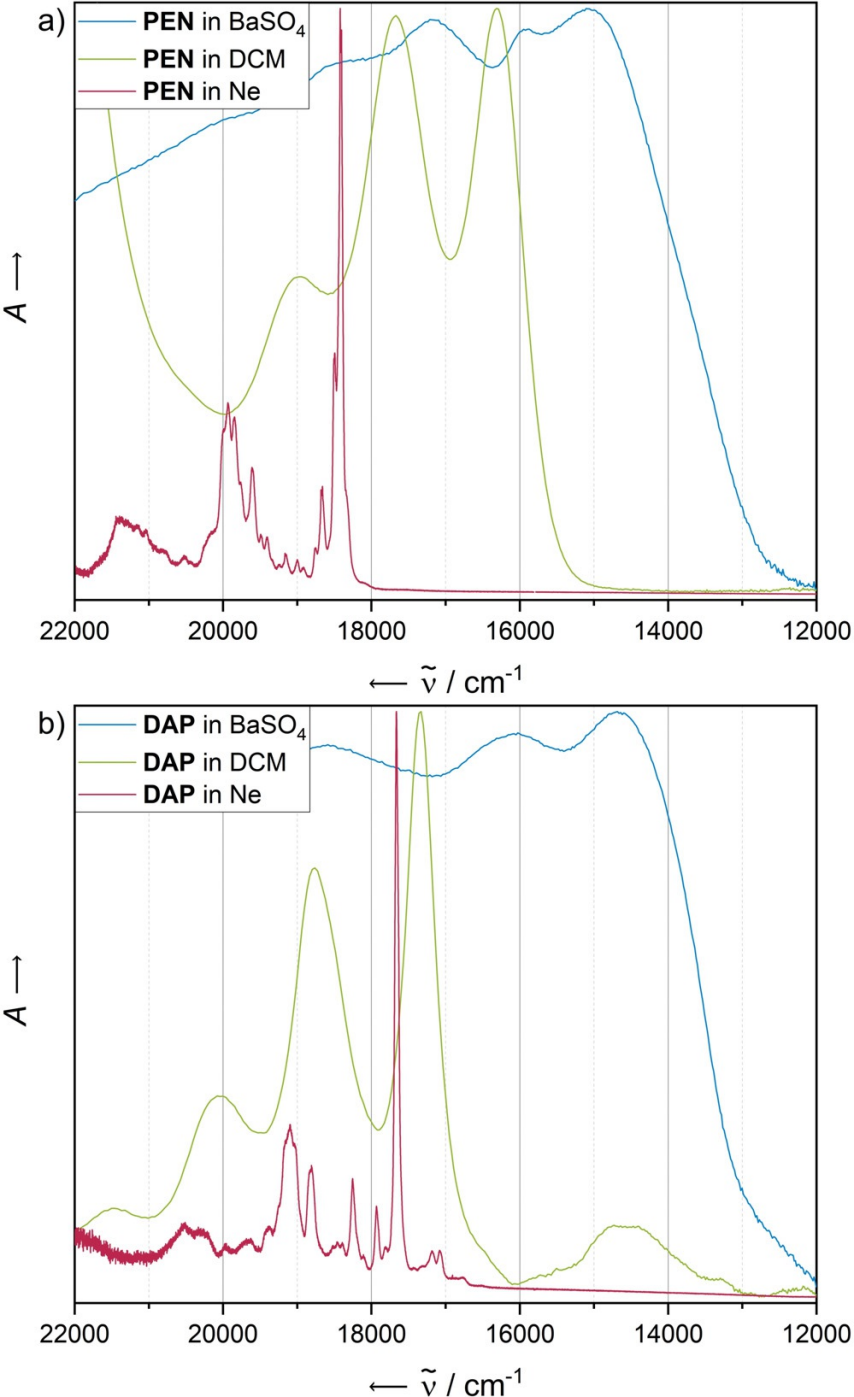
Comparison of normalised UV/Vis spectra of a) **PEN** and b) **DAP** obtained with different methods. Green: transmittance of a 10^−5^ m (**PEN**) or saturated (**DAP**) solution in dichloromethane. Blue: diffuse reflectance spectrum for the solid material embedded in a BaSO_4_ matrix; the absorbance has been calculated as −log(reflectance). Red: visible spectrum of the isolated acene trapped in solid Ne at 4 K.

We have conducted comparative studies of UV/Vis measurements using three different methods: common transmittance measurements in solution, solid‐state diffuse reflectance measurements of the respective acene in a matrix of BaSO_4_ (1:5) and optical characterisation of the acene trapped in solid Ne at 4 K. All absorbance spectra are summarised in Figure 1; Table 1 summarises the most important absorbance bands of both species.

**Table 1:** Comparison of the most dominant experimental electronic transition energies ω of **PEN** and **DAP** recorded in solid Ne matrices at 4 K, as a solution in dichloromethane (DCM) at room temperature and as a solid in a matrix of BaSO_4_ (1:5) at room temperature.

ω(**PEN**)				ω(**DAP**)		
Ne	DCM	BaSO_4_		Ne	DCM	BaSO_4_
18327					≈14528^[b]^	
18410	16313	15105		16783^[a]^		
18495				17070^[a]^		
18661				17181^[a]^		
18753				17664	17331	14684
19602				17933		
19772				18254		
19844				18808		
19930	17667	15923		19095	18762	16077
19986				20523	20040	
21420	18975	17182			21505	18587

[a] Additional bands below the first intensive transition assigned to dimeric species. [b] Broad, red‐shifted bands appearing in solution only and presumably arising from solvent‐driven aggregation or microcrystalline residuals.

For both substances, the solid‐state spectrum (shown in blue) is the most red‐shifted, exhibiting an absorbance edge characteristic for semiconductor materials.

The solution‐based absorbance spectra recorded in dichloromethane (green curves) show a distinct vibronic progression starting at 16 310 cm^−1^ for **PEN** and 17 330 cm^−1^ for **DAP**. These progression patterns are reproduced in the spectra of the matrix‐isolated pentacenes (red curves). However, the rigid character of the matrix combined with the low concentrations used gives much sharper bands. The spectra reveal a detailed fine structure that is not accessible with standard UV/Vis methods due to diverse band broadening mechanisms such as interactions with the solvent, fluctuations in the molecular structure or aggregation. The latter might be the reason for the appearance of a very broad band, red shifted to the first intensive transition, around 14 500 cm^−1^ in the solution‐based vis spectrum of **DAP**.

### Pentacene

**Figure 2:**
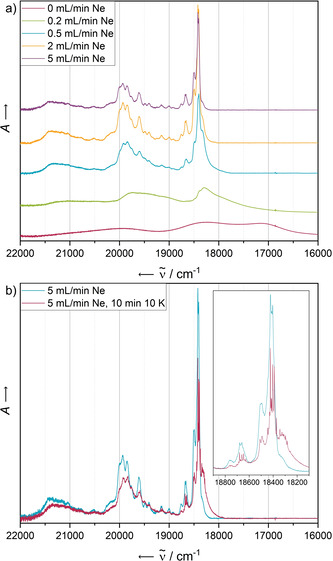
a) Electronic absorbance spectra of **PEN** in solid Ne after deposition for 5 min with a deposition rate of 0.38 Hz s^−1^. The concentration of the analyte has been screened by variation of the Ne flow rate in the range of 0 mL min^−1^ (leading to a non‐diluted solid) to 5 mL min^−1^. b) Electronic absorbance spectra of **PEN** in solid Ne after deposition for 5 min with a neon flow of 5 mL min^−1^ and a deposition rate of 0.38 Hz s^−1^ (blue) and after annealing at 10 K for 10 min (red). Both spectra have been recorded at 4 K.

Following established procedures,^[44]^ we varied the concentration of the analyte by regulating the flow rate of the matrix gas. An increasing neon flow rate leads to a decreasing concentration of the analyte in the resulting matrix. The concentration‐dependent electronic absorbance spectra of pentacene (**PEN**) trapped in matrices of solid neon at 4 K are shown in Figure 2 a.

The absorbance of the pure solid exhibits the most red‐shifted, broadest bands. With decreasing concentration, the absorbance bands are blue‐shifted, sharpened and structured. The most diluted matrices exhibit a vibronically resolved fine structure with a first absorbance maximum at 18 410 cm^−1^.

These data correspond well with previously reported studies on pentacene matrices in various matrix materials, which assigned this band to a 1^1^B_2u_←X^1^A_g_ transition.^[45]^ Our additional concentration‐dependent spectra show as the only difference in the absorbance of the two most diluted matrices a decrease of the intensity of a shoulder red‐shifted with respect to the first intense transition around 18 330 cm^−1^ with an increased flow of neon during deposition. This band can therefore most likely be assigned to a dimeric species whose formation is statistically suppressed when using a higher matrix gas flow. This explanation is supported by a complementary annealing experiment, which by a temporary increase of the particle mobility of the matrix should drive the statistical distribution of species of the analyte towards the formation of energetically favoured, aggregated states such as dimers. After keeping the temperature of the matrix for 10 min at 10 K, the aforementioned red‐shifted band is gaining in relative intensity and appears much more pronounced (Figure 2 b), hinting therefore at the formation of dimers, in line with the results of the concentration‐dependent experiments. At 10 K, the neon atoms are near the sublimation point and very mobile, allowing thermodynamically favoured dimerisation processes to occur. In principle, the formation of large aggregates is also possible, but in highly diluted matrices dimers are much more likely formed than higher aggregates. Additional experiments in which the matrix was heated up stepwise showed complete sublimation of the neon atoms at 12 K (causing a temporary drop of the background pressure in the matrix chamber), leading to a similar spectrum as obtained by deposition in the absence of matrix gas (see Supporting Information, Figure S5).

**Figure 3:**
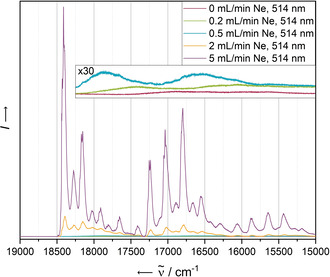
Fluorescence spectra of **PEN** in solid Ne recorded at 4 K with an excitation wavelength of 514 nm after deposition for 5 min with a deposition rate of 0.38 Hz s^−1^. The concentration of the analyte has been screened by variation of the Ne flow rate in the range of 0 mL min^−1^ (leading to an undiluted solid) to 5 mL min^−1^.

While the electronic absorbance spectra of the two most diluted matrices do not display further significant differences, the overall fluorescence intensity significantly decreases in the more concentrated samples, independent from the excitation wavelength (see Figure 3 for an excitation wavelength of 514 nm). As the drastic loss of intensity is observable throughout the entire emission spectrum and not only at higher energies, at which it narrowly overlaps with the absorbance bands, a concentration‐dependent quenching as a result of the absorbance of newly emitted photons by surrounding molecules does not seem to be the predominant quenching mechanism. Instead, this observation may be explained by a singlet fission mechanism populating triplet states that undergo a different, radiationless relaxation mechanism.^[46]^ Previous work on substituted pentacene dimers,^[47]^ covalently linked dimeric species of substituted pentacene^[48]^ and *N*‐heteropentacene^[49]^ have demonstrated the ability of acenes to undergo efficient singlet fission relaxation pathways in solution.

**Figure 4:**
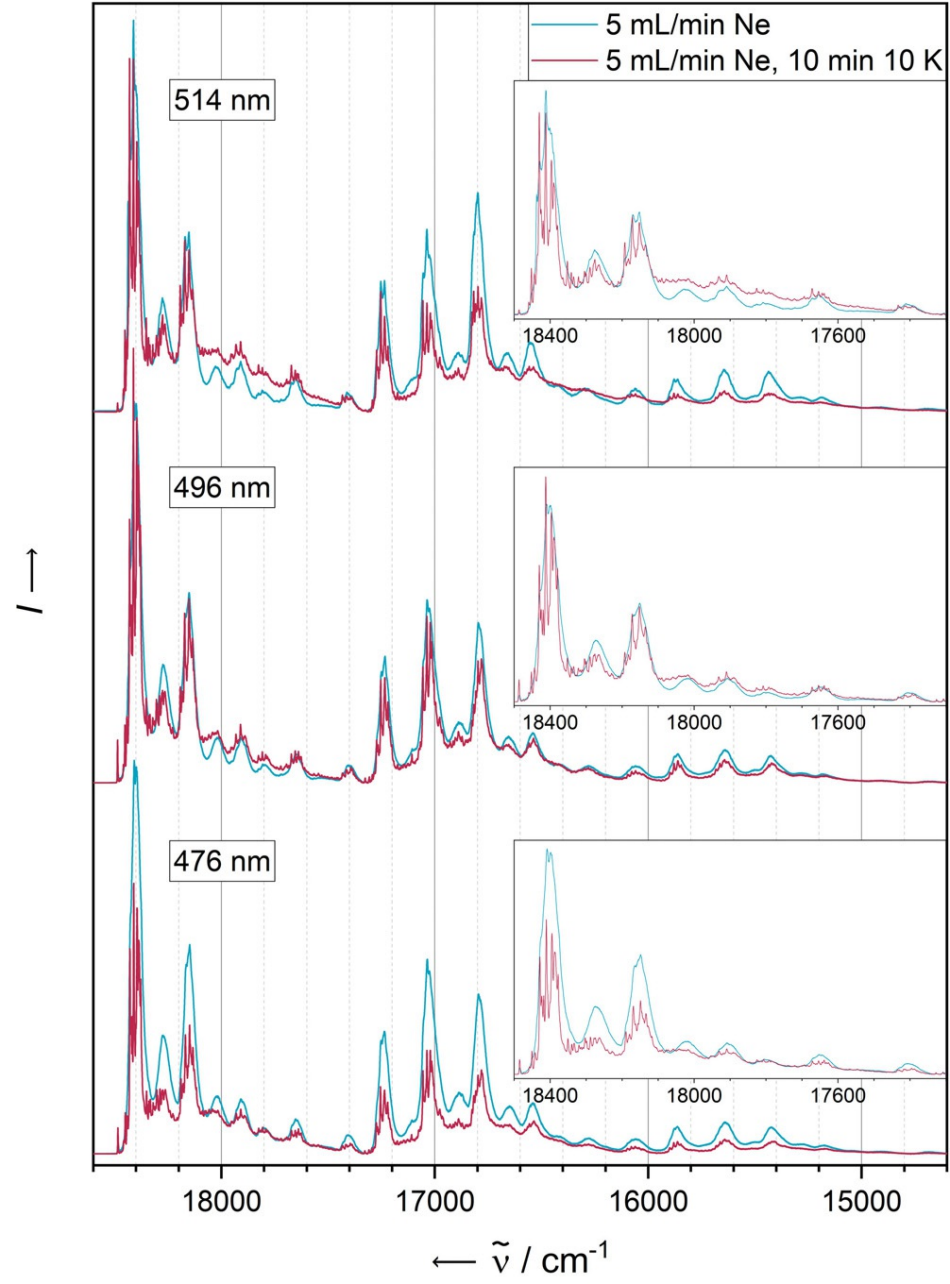
Fluorescence spectra of **PEN** in solid Ne recorded with different excitation wavelengths after deposition for 5 min with a neon flow of 5 mL min^−1^ and a deposition rate of 0.38 Hz s^−1^ (blue) and after annealing at 10 K for 10 min (red). All spectra have been recorded at 4 K.

Figure 4 displays the fluorescence spectra resulting from the most diluted matrix recorded at different excitation wavelengths. In addition to the excitation‐independent overall structure, we observe a fine structure of the emission bands, slightly more pronounced in the case of the lowest‐energy excitation wavelength of 514 nm.

Particular attention with respect to aggregation should be paid to the photophysical behaviour of **PEN** after annealing. The electronic absorbance and emission spectra before and after annealing at 10 K for 10 min are shown in Figure 2 b and Figure 4, each recorded at 4 K. Quenching of the most energetic emission band system after annealing is only observed in the case of the highest‐energy excitation wavelength of 476 nm, suggesting a different relaxation mechanism to be populated compared to lower‐energy excitation with 496 nm or 514 nm light, for which the S_1_→S_0_ transition seems to be the preferred relaxation route.

On the other hand, the second most energetic emission signal exhibits an intensity loss after annealing using the highest (476 nm) and lowest energy (514 nm) excitation wavelengths, but not for the mid‐energy 496 nm excitation wavelength.

All fluorescence signals sharpen after annealing of the matrices (insets of Figure 4). Features already observable before annealing afterwards appear as sharp distinct signals with FWHMs of less than 5 cm^−1^. Additionally, all signals are split into a manifold, independent of the excitation energy. Considering the unaltered positions of the bands before and after annealing, it seems likely that all bands are already present in the matrices after deposition, but detectable as their envelope only due to their larger FWHM. A possible explanation for the detection of such an elevated number of emission bands with spacings as low as 20–30 cm^−1^ is a matrix effect. The slightly inhomogeneous encapsulation of the analyte molecules into the hexagonal densely packed neon atom lattice creates different lattice sites with slightly different interaction energies of the analyte with the surrounding medium. The band sharpening may then be a result of the occupation of more defined lattice sites after the reorganisation of the matrix upon increasing the particle mobility during the annealing. A related effect may be structural distortion that is imposed during the co‐deposition with the noble gas—pentacene has a relatively large backbone that allows easy deformation.

**Figure 5:**
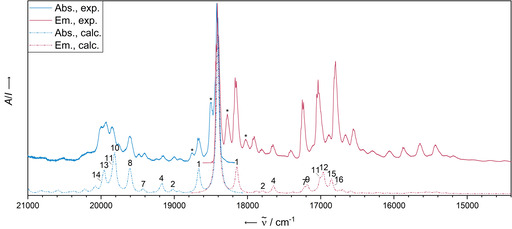
Comparison of experimental (Ne matrix at 4 K after deposition for 5 min at a deposition rate of 0.38 Hz s^−1^ and a neon flow of 10 mL min^−1^) and calculated (B3LYP‐D3BJ/def2‐TZVP) vibrationally resolved electronic absorbance and emission spectra of **PEN**. The computational spectra were blue‐shifted by 2160 cm^−1^ (absorbance) and 2153 cm^−1^ (fluorescence) to match the 0–0 transition in the experimental data. The most dominant vibrational modes are numbered and assigned in Table 3 (see Supporting Information for a full list). Asterisks denote some of the bands that cannot be reproduced by single molecule simulations.

Figure 5 shows the experimental absorbance and emission spectra of **PEN** alongside with the calculated spectra including the assignment of the most intense bands to the underlying vibrational modes. The optical spectra obtained from the Ne matrix can predominantly be attributed to the monomer as shown by comparison with the simulated vibrationally resolved electronic spectra (Table 2 for the contributing normal modes). The first calculated mode of the vibrational progression, with an energy as low as 265 cm^−1^, corresponds to a symmetric stretching mode (*ν*_s_) of the molecule. The mode with the largest normal coordinate displacement and a shift of 1410 cm^−1^ is most adequately described as scissoring [*δ*(H_2_)] of the hydrogen atoms. However, the experimentally obtained absorbance and fluorescence spectra do not entirely match the theoretically obtained ones. Some bands are not represented in the simulations (highlighted by asterisks in Figure 5).

**Table 2:** Assignment *A* of the nine normal modes (all totally symmetric, a_g_) with the respective energy *E* and the largest normal coordinate displacement *D* with leading contributions to the shape/vibrational progression of the vibrationally resolved electronic absorbance and fluorescence spectra of **PEN**.

Absorbance					Fluorescence			
*A*	Normal mode	*E*/cm^−1^	*D*		*A*	Normal mode	*E*/cm^−1^	*D*
1	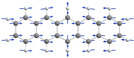	265.49	0.55		1	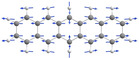	264.31	0.58
2	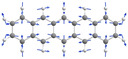	616.86	0.21		2	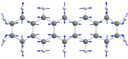	615.21	0.16
4	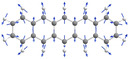	767.44	0.34		4	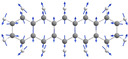	764.59	0.33
7	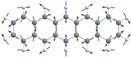	1186.08	0.30		7	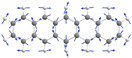	1184.33	0.27
8	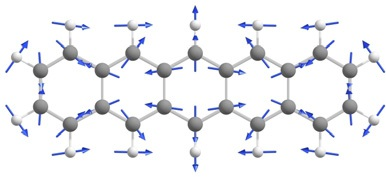	1209.83	0.49		9	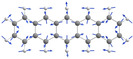	1229.39	0.34
10	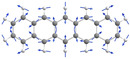	1409.67	0.61		11	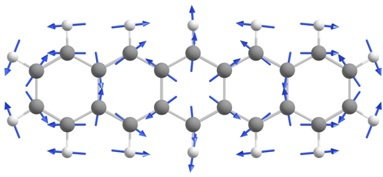	1401.24	0.43
11	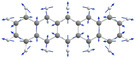	1433.85	0.37		12	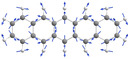	1446.30	0.53
13	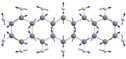	1555.90	0.46		15	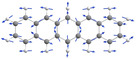	1555.68	0.45
14	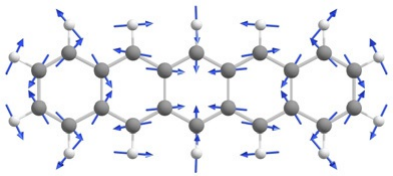	1576.49	0.23		16	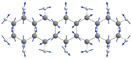	1572.21	0.17

Interestingly, comparing the experimental spectra reveals that the same additional bands are not part of any obvious absorbance—fluorescence band pair. On the other hand, a superposition with a second but shifted monomer spectrum results in the reproduction of the experimental spectra of pentacene (see Figure S4). A similar occurrence in the matrix spectra was previously observed in the case of tetracene and assigned to van der Waals dimers.^[44]^ We also assign herein the additional bands to **PEN** dimers, possibly in the form of H aggregation due to the blue shift in the absorbance, for which we are however not yet able to provide a matching structural proposal due to the computational demands.

### 6,13‐Diazapentacene

**Figure 6:**
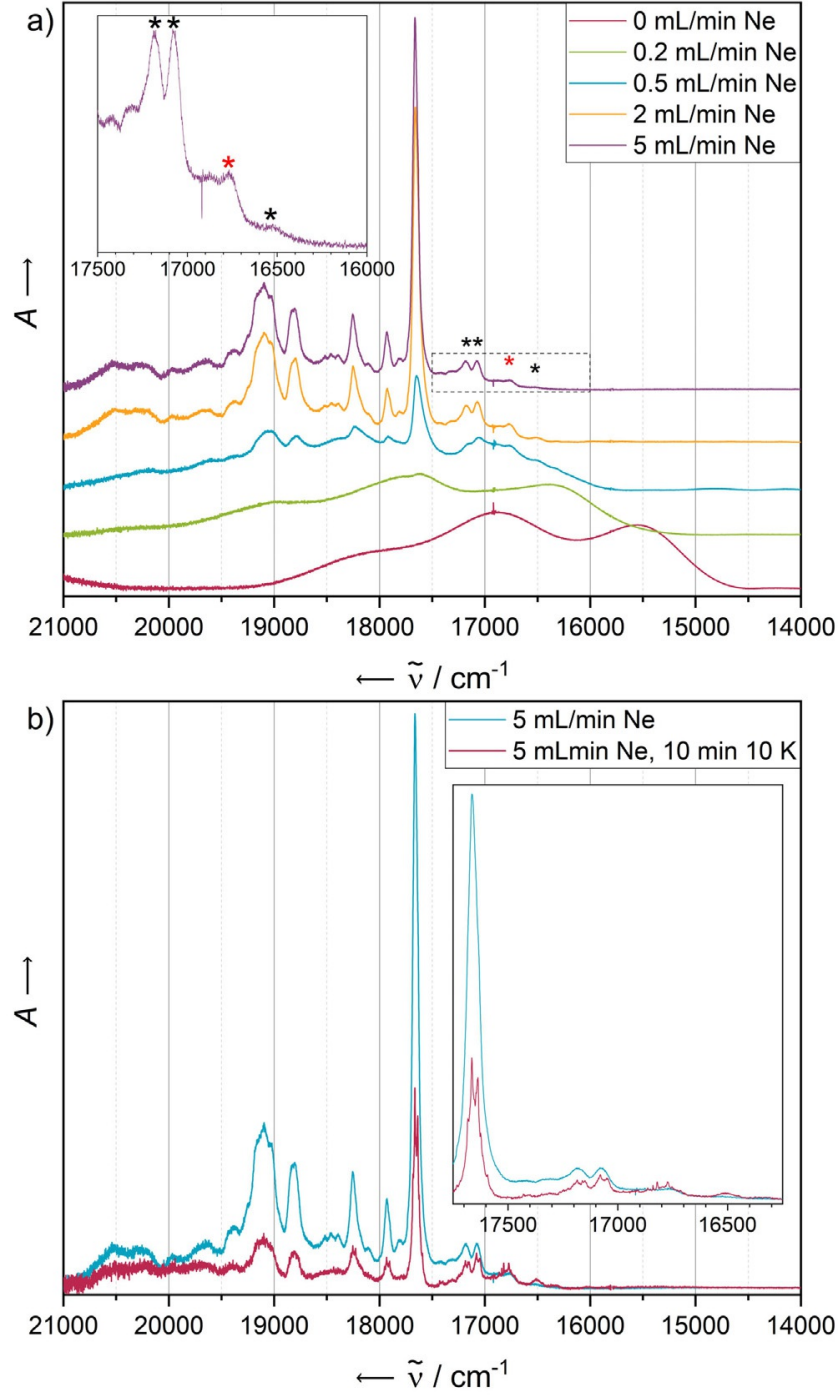
a) Electronic absorbance spectra of **DAP** in solid Ne after deposition for 5 min. with a deposition rate of 0.19 Hz s^−1^. The concentration of the analyte has been screened by variation of the Ne flow rate in the range of 0 mL min^−1^ (leading to an undiluted solid) to 5 mL min^−1^. Asterisks denote bands assigned to dimeric species. Red asterisk has a corresponding band in the fluorescence spectrum after annealing (see Figure 8). b) Electronic absorbance spectra of **DAP** in solid Ne after deposition for 5min with a neon flow of 5 mL min^−1^ and a deposition rate of 0.19 Hz s^−1^ (blue) and after annealing at 10 K for 10 min (red). Both spectra have been recorded at 4 K.

**Figure 7:**
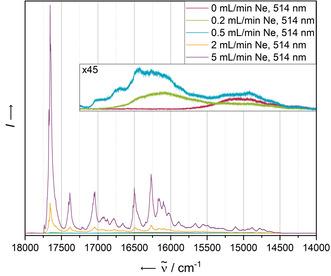
Fluorescence spectra of **DAP** in solid Ne recorded with an excitation wavelength of 514 nm after deposition for 5 min with a deposition rate of 0.19 Hz s^−1^. The concentration of the analyte has been screened by variation of the Ne flow rate in the range of 0 mL min^−1^ (leading to an undiluted solid) to 5 mL min^−1^.

6,13‐Diazapentacene (**DAP**) was subjected to the same concentration‐dependent experiment (absorbance in Figure 6 a, fluorescence in Figure 7). A systematic blue shift accompanied by a band narrowing upon decreasing the concentration and a growth of a most intensive absorbance signal at 17 664 cm^−1^ results. In contrast to **PEN**, additional red‐shifted absorbance bands appear (highlighted by asterisks).

Upon annealing of the matrix (Figure 6 b), the bands systematically disintegrate into sharp signals with small spacings as for **PEN**, including the red‐shifted smaller bands, as a result of a structural relaxation induced by the increase of particle mobility. We observe quenching of the absorbance spectrum with the exception of the additional red‐shifted bands (inset of Figure 6 b).

**Figure 8:**
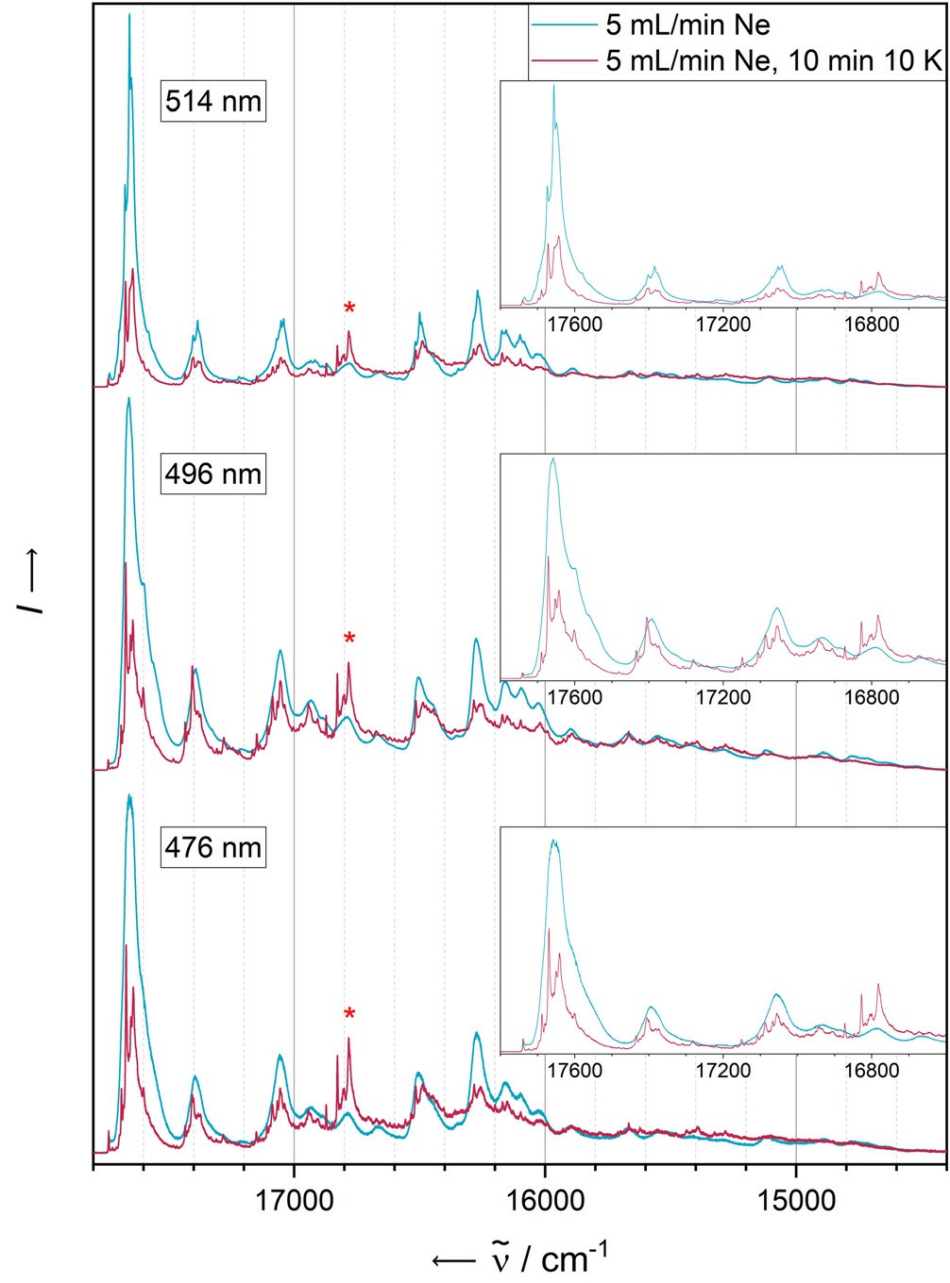
Fluorescence spectra of **DAP** in solid Ne recorded with an excitation wavelength of 514 nm after deposition for 5 min with a deposition rate of 0.19 Hz s^−1^. The concentration of the analyte has been screened by variation of the Ne flow rate in the range of 0 mL min^−1^ (leading to an undiluted solid) to 5 mL min^−1^. Red asterisks denote a band assigned to a dimeric species having a corresponding band in the absorbance spectra (see Figure 6).

The comparison of the fluorescence intensities obtained with different excitation wavelengths before and after annealing of the most diluted matrix (Figure 8) gives a significantly different picture as in the case of the unsubstituted **PEN**. In the case of **DAP**, pronounced quenching of the intensity to roughly one half is observed for all applied excitation wavelengths. This is in line with previous studies on substituted pentacene derivatives with and without nitrogen substitution in the molecular backbone, showing some evidence for an accelerated and therefore more competitive singlet‐fission relaxation mechanism arising from stronger intermolecular interactions.^[50]^

Note that one single band of the emission spectrum at 16 783 cm^−1^ (red‐shifted by about 880 cm^−1^ with respect to the 0–0 transition) is crucially gaining in intensity after annealing. This signal will be discussed in more detail below.

**Figure 9:**
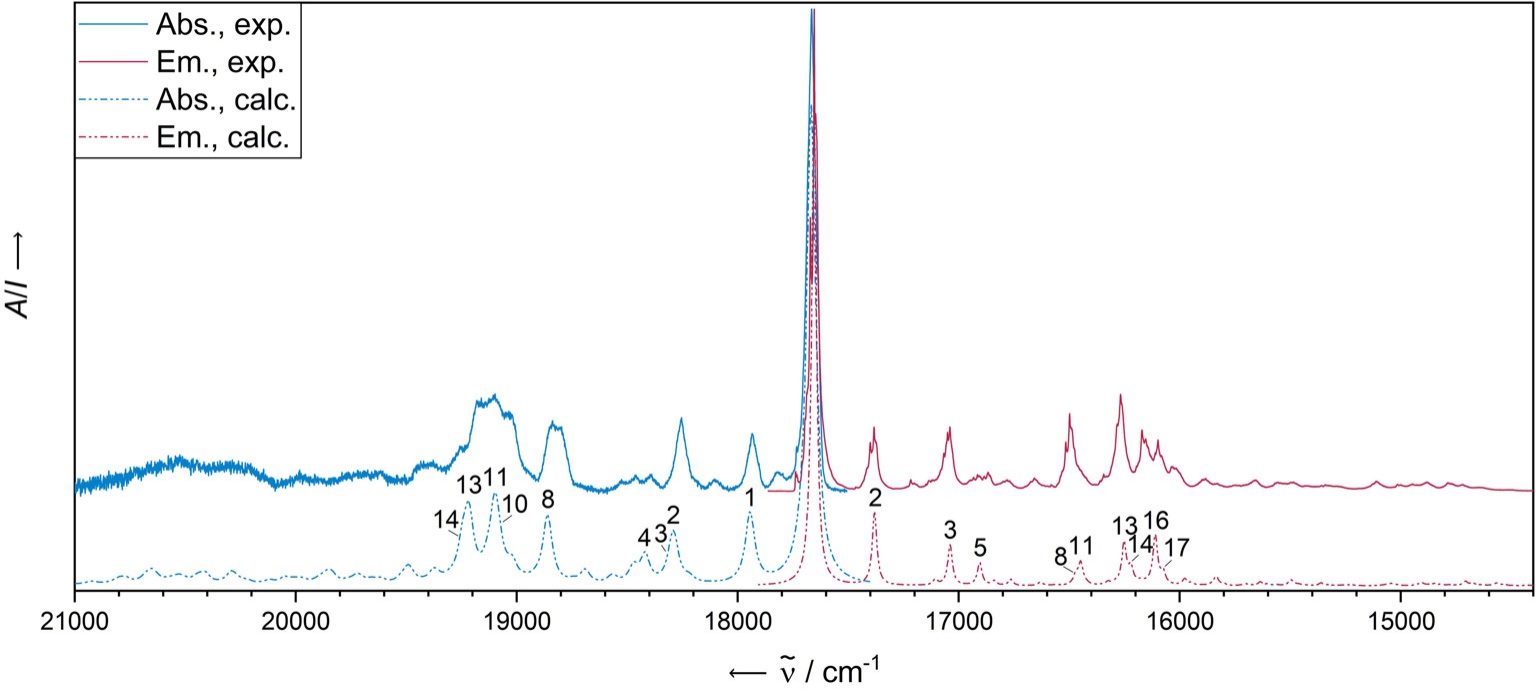
Comparison of the experimental (Ne matrix at 4 K after deposition for 5 min at a deposition rate of 0.19 Hz s^−1^ and a neon flow of 10 mL min^−1^) and calculated (B3LYP‐D3BJ/def2‐TZVP) vibrationally resolved electronic absorbance and emission spectra of **DAP**. The computational spectra were shifted by 2080 cm^−1^ (absorbance) and 2051 cm^−1^ (fluorescence) to match the 0–0 transition in the experimental data. The most dominant vibrational modes are numbered and assigned in Table 3 (see Supporting Information for a full list).

The simulation of the vibrationally resolved electronic absorbance spectrum of a single **DAP** molecule without inclusion of environmental effects corroborates the experimental capability to obtain the electronic absorption spectrum of a single unperturbed **DAP** molecule. The vibrational progression is produced by an excitation into the first excited singlet state (S_1_) (see Figure 9 for the spectrum and Table 3 for major contributing modes, respectively). The first calculated mode of the vibrational progression, with an energy as low as 276 cm^−1^, corresponds to a symmetric stretching mode (*ν*_s_) of the molecule. The mode with the largest normal coordinate displacement and a shift of 1193 cm^−1^ is most adequately described as a scissoring [δ(H_2_)] of the hydrogen atoms. The S_1_ state is characterised as the ^1^L_w_ state, polarised along the short axis and having a less correlated electron‐hole pair compared to its L_s_ state (S_4_) and its higher correlated exciton.[^51^, ^52^] Similar to the electronic absorbance spectrum, most features of the experimental fluorescence spectrum are equally well reproduced by the simulated single unperturbed molecule spectrum (Figure 9, Table 3).

**Table 3:** Assignment *A* of the nine normal modes (all totally symmetric, a_g_) with the respective energy *E* and the largest normal coordinate displacement *D* with leading contributions to the shape/vibrational progression of the vibrationally resolved electronic absorbance and fluorescence spectra of **DAP**.

Absorbance					Fluorescence			
*A*	Normal mode	*E*/cm^−1^	*D*		*A*	Normal mode	*E*/cm^−1^	*D*
1	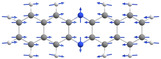	275.77	0.54		2	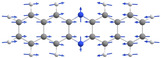	274.57	0.56
2	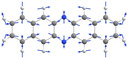	619.12	0.38		3	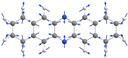	616.22	0.43
3	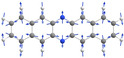	631.95	0.27		5	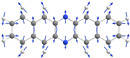	750.19	0.33
4	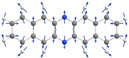	751.31	0.33		8	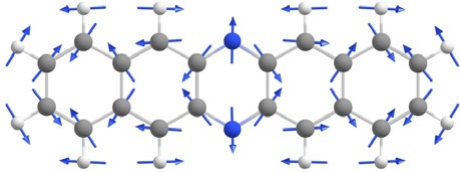	1185.46	0.23
8	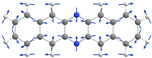	1192.78	0.50		11	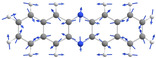	1207.47	0.33
10	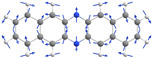	1414.65	0.32		13	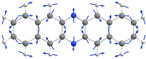	1403.57	0.48
11	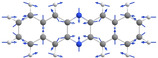	1433.41	0.49		14	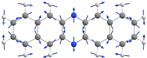	1434.47	0.29
13	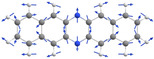	1548.41	0.49		16	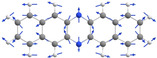	1544.82	0.53
14	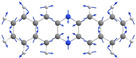	1577.58	0.35		17	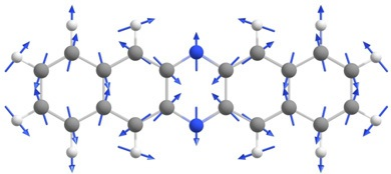	1580.34	0.26

**Figure 10:**
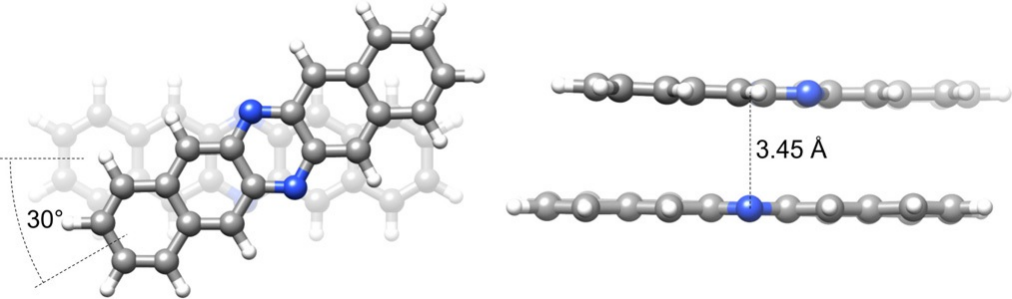
Lowest‐energy minimum structure of the **DAP** dimer with a binding energy of 68.1 kJ mol^−1^, according to ωB97X‐D3/def2‐TZVPP calculations.

However, some observed red‐shifted bands (shifted by a few hundred cm^−1^, highlighted by asterisks in Figure 6 a) are not reproduced by the single‐molecule spectrum and could be assigned to the van der Waals dimer. Comparing the excitation energy and oscillator strength of the S_1_ state of the energetically lowest minimum dimer structure found within a tentative search (see Figure 10 and computational details) with the S_1_ of the respective monomer indicates a decreased excitation energy from 22 036 cm^−1^ for the monomer to 21 480 cm^−1^ for the dimer, as well as a decreased oscillator strength (dimer: 0.003, monomer: 0.111). Thus, the conformational space of dimers produces structures displaying red‐shifted low‐intensity bands with respect to the monomer. The calculated red shift of 556 cm^−1^ is in fair agreement with the experimental red shift of 880 cm^−1^ corresponding to the coupling constant *J* of the dimer. This assignment is supported by the observation of the fluorescence band at 16 783 cm^−1^, appearing after annealing, that has an exact analogue in the absorbance spectrum. We assign this band to a S_1_←S_0_ transition of a non‐covalent dimer void of Stokes shift, in similarity to the monomer spectra.

## Conclusions

In summary, we have demonstrated the ability of the matrix‐isolation technique to produce high‐resolution optical spectra (electronic excitation and emission) of monomeric pentacene derivatives well correlated with quantum‐chemical calculations on unperturbed single pentacene and 6,13‐diazapentacene molecules free of environmental effects. The main vibrational modes leading to the progression patterns were assigned. The spectra give valuable insights into important vibrational modes of the ground state as well as the excited state.

For both molecules, additional bands appear that could be assigned to van der Waals dimers on the basis of the response to variations in the concentration and to annealing experiments as well as quantum‐chemical calculations. For pentacene, these bands in the absorbance and the fluorescence spectra that do not belong to the monomer S_1_ state are assigned to H‐type dimerisation leading to a blue shift in the dimer absorbance (by 100 cm^−1^) and a Stokes shift as a consequence of strong excitonic coupling. 6,13‐Diazapentacene exhibits additional absorbance bands red‐shifted to the ^1^L_w_ transition gaining in intensity upon annealing. In particular, a band red‐shifted by about 880 cm^−1^ from the 0–0 transition of the monomer is assigned to the 0–0 transition of the S_1_←S_0_ excitation of a van der Waals dimer, supported by a significant increase of a fluorescence signal at similar energy upon annealing. Based on quantum‐chemical calculations, a dimer structure is suggested with a 0–0 transition that is in fair agreement with the experimental value. Hence, in this work we provide for the first time spectroscopic evidence for the dimers of pentacene and 6,13‐diazapentacene, providing further useful insights into the complex optical properties of polyaromatic aggregates.^[53]^ The change of electronic characteristics upon insertion of sp^2^ nitrogen into the molecular backbone is clearly reflected in the different spectral features of the dimeric species.

## Experimental Section

### Experimental setup

**PEN** was purchased from TCI Europe with a purity of 99.999 % and used without further purification. **DAP** was synthesized according to literature procedures[^54^, ^55^] and purified by recrystallisation from DMSO and sublimation. Matrix isolation experiments were conducted using standard techniques, in this form first presented by Pimentel et al. in 1954^[40]^ and further developed by Andrews, Maier et al.[^56^, ^57^, ^58^, ^59^, ^60^, ^61^, ^62^] We have described the setup of our matrix apparatus in detail elsewhere.^[63]^ Preliminary calibration measurements have been conducted using a separate quartz apparatus to determine the evaporation rates as a function of the applied voltage (see Supporting Information for a more detailed description). The evaporation of the substances has been carried out with a water‐cooled Knudsen‐type effusion cell containing a graphite tube inside a ceramic unit which was heated by applying a defined voltage to a surrounding Ta coil. The substances were co‐deposited with Ne (Air Liquide, 99.999 %) to create matrices on a Rh‐coated Cu surface which was held at 4 K using a pulse‐tube cooler (Vericold) and a closed‐cycle helium cryostat (Leybold). During deposition, the flow of the Ne gas was kept constant with a flow controller (EL‐FLOW, Bronkhorst). Vis spectra were recorded with a Bruker Vertex 80v spectrometer with a resolution of 1 cm^−1^, with a tungsten lamp, a CaF_2_ beam splitter and a Si diode detector. Fluorescence spectra were obtained with a Symphony II charge‐coupled device (CCD) detector (Horiba) using a binning factor of 1 after excitation with an Ar laser (Innova 90c‐A3; Coherent).

### Computational details

The calculation and subsequent simulation of vibrationally resolved electronic spectra (absorption and fluorescence) were performed using a time‐dependent approach of an independent mode displaced harmonic oscillator model derived by Heller as implemented in the ORCA software package.[^64^, ^65^] Kohn–Sham DFT using the B3LYP functional and the def2‐TZVP basis was used for ground and excited state geometry optimisations with subsequent frequency calculations as well as for the calculation of excited states via linear‐response time‐dependent DFT (TDDFT) with the Tamm–Dancoff approximation employed.[^66^, ^67^, ^68^] Calculations of the **DAP** dimer structures were performed using a starting grid of multiple dimer conformations first optimized on a PBEh‐3c level of theory. The minimum structures were then refined on a ωB97X‐D3/def2‐TZVPP level of theory.

## Conflict of interest

The authors declare no conflict of interest.

## Supporting information

As a service to our authors and readers, this journal provides supporting information supplied by the authors. Such materials are peer reviewed and may be re‐organized for online delivery, but are not copy‐edited or typeset. Technical support issues arising from supporting information (other than missing files) should be addressed to the authors.

Supplementary
