# Supplementary material for: High‐Resolution Electronic Excitation and Emission Spectra of Pentacene and 6,13‐Diazapentacene Monomers and Weakly Bound Dimers by Matrix‐Isolation Spectroscopy
Source: Chemistry. 2020 Dec 22;27(6):2072–81. doi: 10.1002/chem.202003999 (PMC7898606; doi:10.1002/chem.202003999)
Supplement: Supplementary file 1 — Supplementary [file CHEM-27-2072-s001.pdf]

Chemistry–A European Journal

Supporting Information

High-Resolution Electronic Excitation and Emission Spectra of Pentacene and 6,13-Diazapentacene Monomers and Weakly Bound Dimers by Matrix-Isolation Spectroscopy

Jean Thusek,[a] Marvin Hoffmann,[b] Olaf Hübner,[a] Stefan Germer,[a] Hendrik Hoffmann,[c] Jan Freudenberg,[c] Uwe H. F. Bunz,*[c] Andreas Dreuw,*[b] and Hans-Jörg Himmel*[a]

Table of contents

1. Deposition rate calibration measurements 2

2. Absorbance / fluorescence spectra of DAP monomer and dimer 4

3. Comparison of experimental absorbance / fluorescence spectra with calculated data 5

4. Stepwise full evaporation of Ne from a matrix containing pentacene (PEN) 6

5. Calculated normal modes of monomers 7

   5.1. Pentacene (PEN) 7

   5.2. 6,13-Diazapentacene (DAP) 8

6. Cartesian coordinates (in Å) of optimised structures 9

   6.1. Pentacene (PEN) 9

   6.1.1. Ground state equilibrium structures 9

   6.1.2. Excited state (S_1) equilibrium structures 10

   6.2. 6,13-Diazapentacene (DAP) 11

   6.2.1. Ground state equilibrium structures 11

   6.2.2. Excited state (S_1) equilibrium structures 12

   6.2.3. Dimer ground state equilibrium structure 13

1. Deposition rate calibration measurements

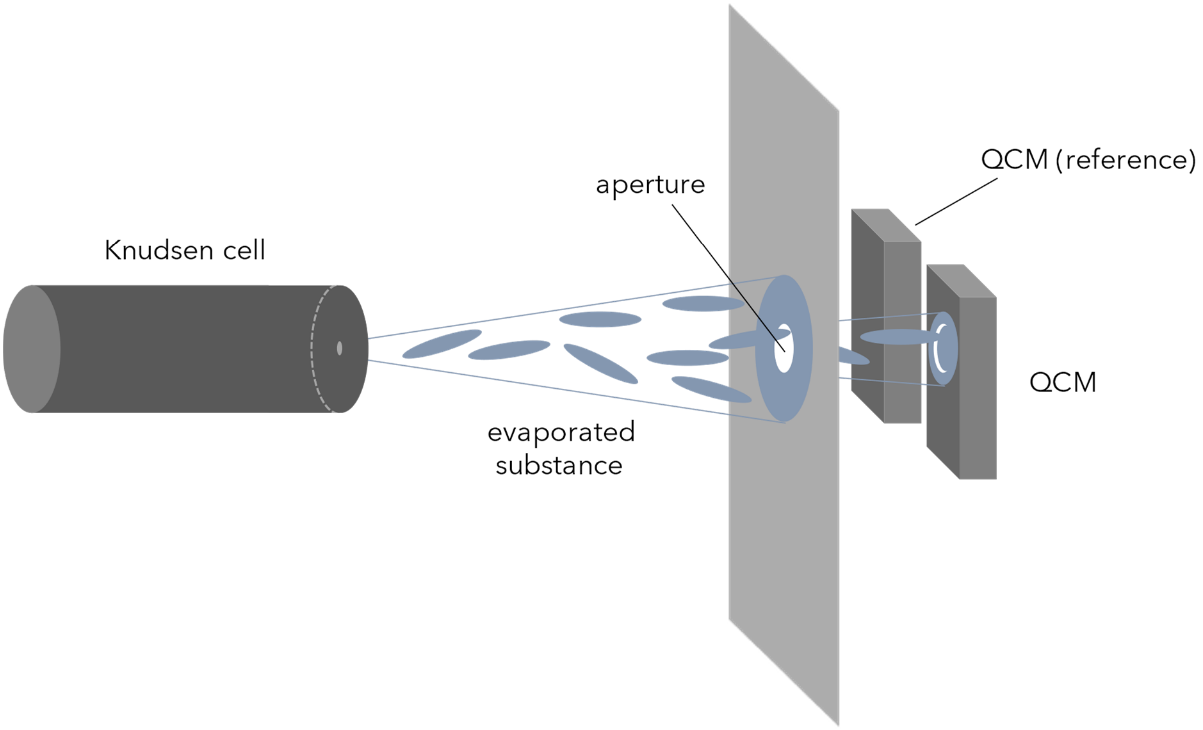

Figure S1. Schematic drawing of the quartz crystal microbalance (QCM) setup for preliminary evaporation calibration measurements. The substance is heated using the same Knudsen effusion cell as for matrix experiments (heating unit: ceramic oven surrounded by Ta coil to which voltage is applied, not shown for the sake of clarity). The substance beam is reduced by a plate containing an aperture and afterwards finally deposited on a QCM. The effective deposition rate is obtained as the increase of the QCM frequency in $Hz{\cdot}s^{-1}$ relative to a shielded quartz, which correlates with the mass of the deposited thin film via $\frac{\Delta f}{f} = -\frac{\Delta d}{d} = -\frac{\Delta m}{\varrho \cdot F \cdot d}$ where $\frac{\Delta f}{f}$ is the eigenfrequency change of the QCM plate with the area F, the density ϱ and thickness d increased by Δd with a substance of the mass Δm. A detailed description can be found in Sauerbrey, G. Verwendung von Schwingquarzen zur Wägung dünner Schichten und zur Mikrowägung. *Z. Physik* 155, 206-222 (1959). https://doi.org/10.1007/BF01337937.

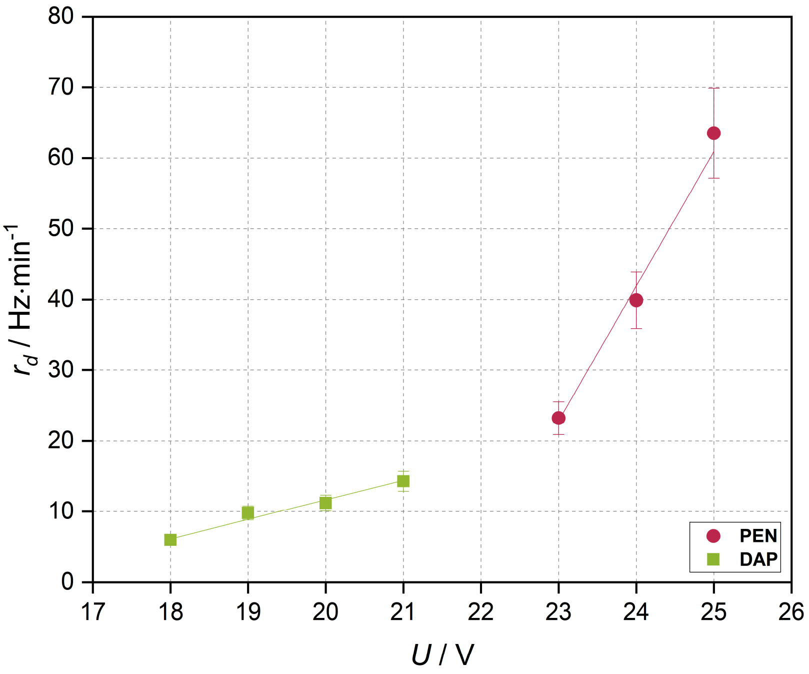

Figure S2. Dependence of the deposition rate, r_d, on the applied voltage for PEN and DAP.

2. Absorbance / fluorescence spectra of DAP monomer and dimer

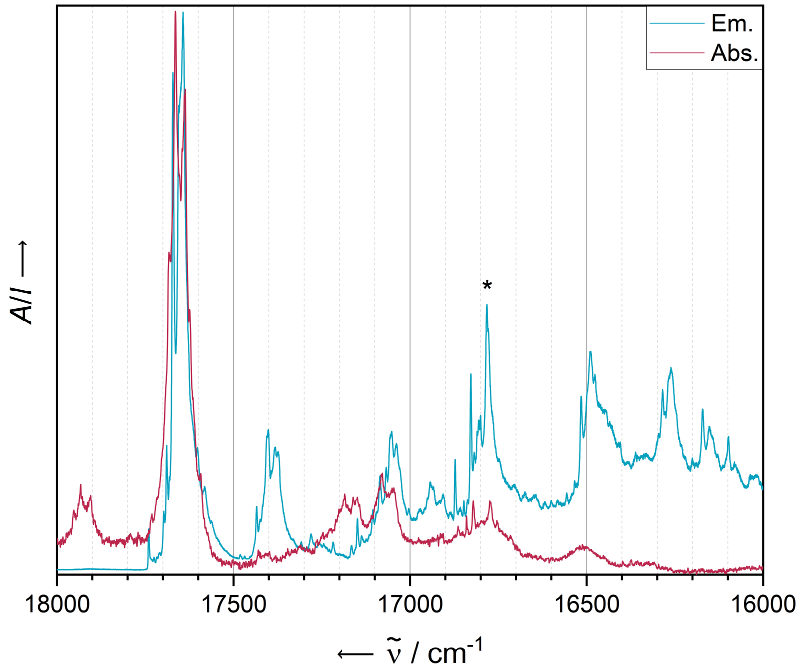

Figure S3. Normalised absorbance/fluorescence spectra of DAP recorded at 4 K after annealing at 10 K for 10 min. The band highlighted by asterisk is assigned to the 0–0 transition of the van der Waals dimer (see main text for a more detailed description).

3. Comparison of experimental absorbance / fluorescence spectra with calculated data

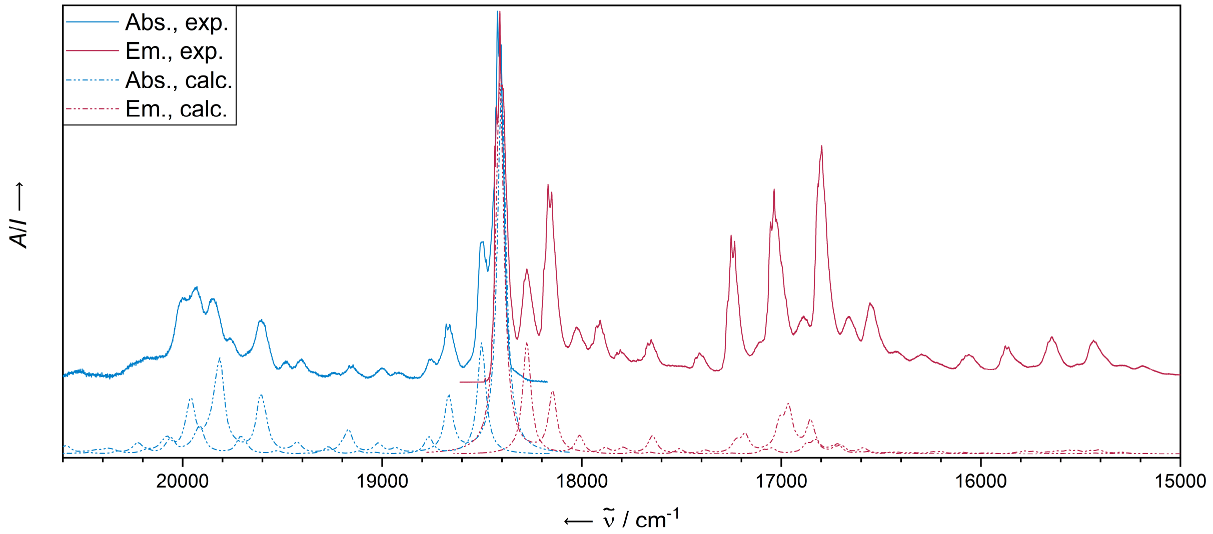

Figure S4. Comparison of experimental (Ne matrix at 4 K after deposition for 5 min at a deposition rate of 0.38 Hz·s^{-1} and a neon flow of 10 ml·s^{-1}) and calculated (B3LYP-D3BJ/def2-TZVP) vibrationally-resolved electronic absorbance and emission spectra of **PEN**. The computational spectra were blue-shifted by 2160 cm^{-1} (absorbance) and 2153 cm^{-1} (fluorescence) to match the 0–0 transition in the experimental data. A superposition of the calculated spectra was created by further blue-shifting the absorbance of +100 cm^{-1} (scaled by 0.3) and blue-shifting the fluorescence by -135 cm^{-1} (scaled by 0.3).

4. Stepwise full evaporation of Ne from a matrix containing pentacene (PEN)

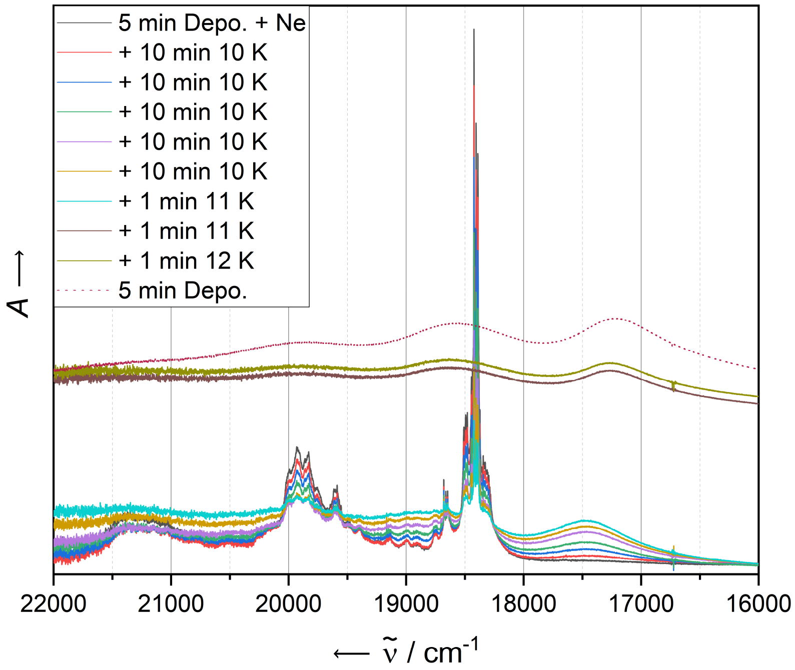

Figure S5. Electronic absorbance spectra of **PEN** in solid Ne after deposition for 5 min with a deposition rate of 0.38 $Hz{\cdot}s^{-1}$ and a Ne flow rate of 5 $ml{\cdot}min^{-1}$. The matrix has been annealed multiple times at 10 K and further at higher temperatures up to 12 K to fully remove the Ne. The spectra show a slow transition to the absorbance spectrum of the pure solid recorded of a sample deposited without any buffer gas.

5. Calculated normal modes of monomers

5.1. Pentacene (PEN)

Table S1. Assignment *A* of the nine normal modes with the respective energy *E*, the symmetry and the largest normal coordinate displacement *D* with leading contributions to the shape/vibrational progression of the vibrationally-resolved electronic absorbance and fluorescence spectra of PEN.

Absorbance					Fluorescence				
A	Normal mode	Symmetry	*E* / cm^{-1}	*D*	*A*	Normal mode	Symmetry	*E* / cm^{-1}	*D*
1	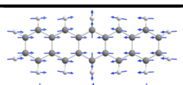	a_g	265.49	0.55	1	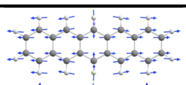	a_g	264.31	0.58
2	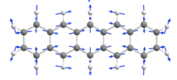	a_g	616.86	0.21	2	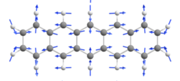	a_g	615.21	0.16
3	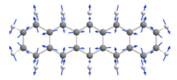	a_g	641.38	0.05	3	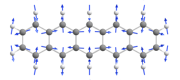	a_g	631.32	0.08
4	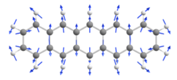	a_g	767.44	0.34	4	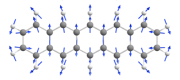	a_g	764.59	0.33
5	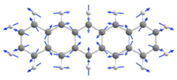	a_g	800.90	0.14	5	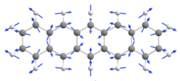	a_g	808.39	0.01
6	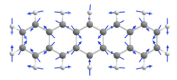	a_g	1020.32	0.18	6	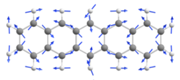	a_g	1047.91	0.02
7	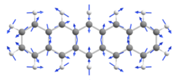	a_g	1186.08	0.30	7	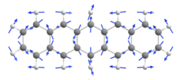	a_g	1184.33	0.27
8	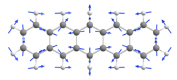	a_g	1209.83	0.49	8	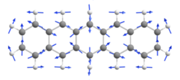	a_g	1219.39	0.01
9	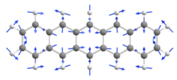	a_g	1338.34	0.12	9	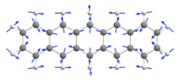	a_g	1229.39	0.34
10	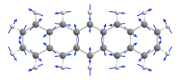	a_g	1409.67	0.61	10	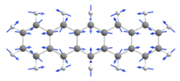	a_g	1330.53	0.10
11	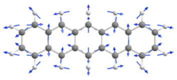	a_g	1433.85	0.37	11	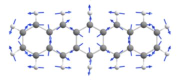	a_g	1401.24	0.43
12	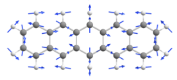	a_g	1497.17	0.11	12	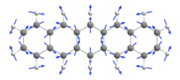	a_g	1446.30	0.53
13	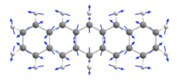	a_g	1555.90	0.46	13	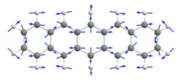	a_g	1512.64	0.05
14	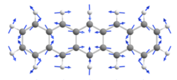	a_g	1576.49	0.23	14	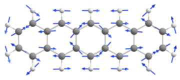	b_{1g}	1554.59	0.01
15	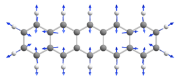	a_g	3190.57	0.01	15	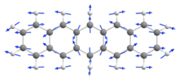	a_g	1555.68	0.45
					16	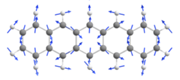	a_g	1572.21	0.17
					17	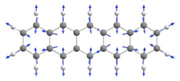	a_g	3195.77	0.01

5.2. 6,13-Diazapentacene (DAP)

Table S2. Assignment *A* of the nine normal modes with the respective energy *E*, the symmetry and the largest normal coordinate displacement *D* with leading contributions to the shape/vibrational progression of the vibrationally-resolved electronic absorbance and fluorescence spectra of **DAP**.

Absorbance					Fluorescence				
A	Normal mode	Symmetry	*E* / cm^{-1}	*D*	*A*	Normal mode	Symmetry	*E* / cm^{-1}	*D*
1	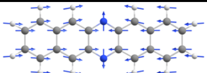	a_g	275.77	0.54	1	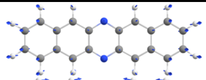	b_{1u}	43.32	0.01
2	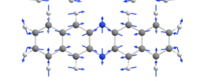	a_g	619.12	0.38	2	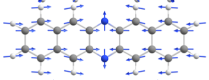	a_g	274.57	0.56
3	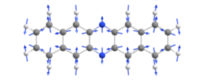	a_g	631.95	0.27	3	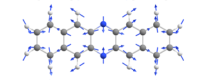	a_g	616.22	0.43
4	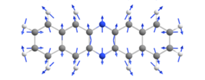	a_g	751.31	0.33	4	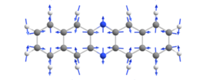	a_g	620.93	0.06
5	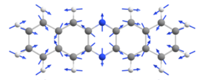	a_g	803.24	0.25	5	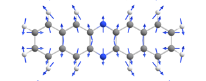	a_g	750.19	0.33
6	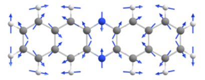	a_g	1019.85	0.19	6	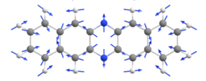	a_g	815.18	0.14
7	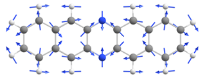	a_g	1187.03	0.13	7	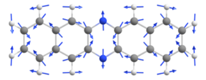	a_g	1047.19	0.03
8	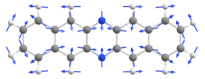	a_g	1192.78	0.50	8	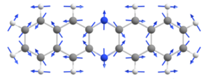	a_g	1185.46	0.23
9	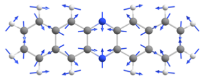	a_g	1351.06	0.24	9	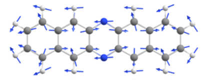	b_{3u}	1204.86	0.01
10	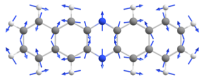	a_g	1414.65	0.32	10	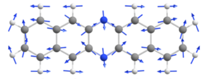	b_{2u}	1206.98	0.06
11	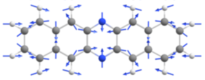	a_g	1433.41	0.49	11	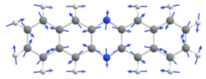	a_g	1207.47	0.33
12	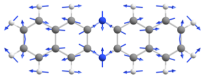	a_g	1495.74	0.03	12	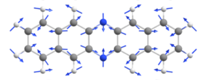	a_g	1331.13	0.13
13	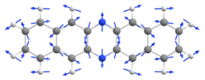	a_g	1548.41	0.49	13	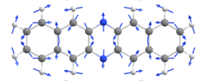	a_g	1403.57	0.48
14	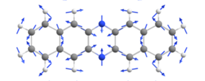	a_g	1577.58	0.35	14	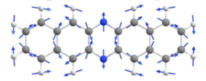	a_g	1434.47	0.29
15	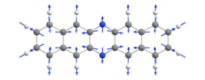	a_g	3180.03	0.01	15	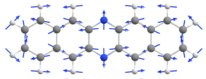	a_g	1512.56	0.08
16	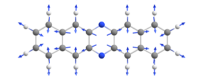	a_g	3192.70	0.01	16	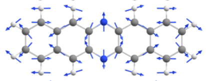	a_g	1544.82	0.53
					17	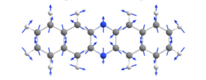	a_g	1580.34	0.26
					18	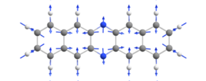	a_g	3175.79	0.01
					19	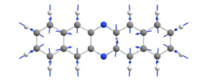	a_g	3198.51	0.01

6. Cartesian coordinates (in Å) of optimised structures

6.1. Pentacene (PEN)

6.1.1. Ground state equilibrium structures

Cartesian coordinates (in Å)

C	1.22093380888524	-0.72561124829086	-0.00001843439977
C	1.22092383191318	0.72603841905024	-0.00001175509622
C	2.45706976959650	1.40338877220244	0.00001646456025
C	3.66215543243647	0.72531462530456	0.00001403410571
C	3.66216999035588	-0.72484731112826	-0.00001135018940
C	2.45709386338199	-1.40293921032049	-0.00001740830325
C	4.92109648492834	-1.40543550517768	-0.00001777107917
C	6.09127929817644	-0.71426084851269	0.00000811966126
C	6.09126843421633	0.71476077758200	0.00003765911324
C	4.92107683671505	1.40591510462071	0.00003709820103
C	-0.00012283776423	-1.40338188340095	-0.00001581873278
C	-0.00013555143960	1.40380117677389	-0.00003363336259
H	2.45740749546211	2.48802951236139	0.00003323817509
H	2.45745208960209	-2.48758054271151	-0.00002562334846
H	4.92044831185763	-2.48927876797397	-0.00003694285874
H	7.03541169476477	-1.24382847308560	0.00000822281856
H	7.03539638873069	1.24433635987505	0.00005594143262
H	4.92041040797236	2.48975796993918	0.00005886757031
C	-1.22118094675330	-0.72563028564647	-0.00003156183093
C	-2.45734033121149	-1.40297212973053	-0.00002112176165
C	-2.45734628039713	1.40335183666968	-0.00005698465365
C	-4.92135104545807	-1.40547668930620	0.00002703202714
C	-3.66242641862979	-0.72488580731423	-0.00000734195616
C	-6.09153038224097	0.71471726676766	0.00002982713889
C	-3.66242611926324	0.72527957460074	-0.00003022508021
C	-4.92134525140169	1.40587967251048	-0.00001537509246
C	-1.22118821528784	0.72602526975599	-0.00004432392247
C	-6.09153400668623	-0.71430456095210	0.00004523499031
H	-7.03566768516650	-1.24387018273418	0.00008654762943
H	-2.45769716583529	-2.48761256032887	-0.00000572438262
H	-4.92069034864143	-2.48931889192216	0.00004446750373
H	-2.45769617437883	2.48799365112398	-0.00006975769726
H	-4.92066628842474	2.48972304922189	-0.00002671405181
H	-7.03565581664367	1.24429654372845	0.00004539399655
H	-0.00011029408316	-2.48791799578665	-0.00001423356009
H	-0.00014103418788	2.48833821173506	-0.00003604756442

6.1.2. Excited state (S_1) equilibrium structures

Cartesian coordinates (in Å)

C	1.22908121892404	-0.72415003132917	-0.00000532675592
C	1.22907005623135	0.72458127806937	-0.00000545602866
C	2.45631598684900	1.39822893680597	-0.00002332905317
C	3.68447746798916	0.72166203033887	-0.00000188812115
C	3.68448401881828	-0.72119284299755	0.00001335859915
C	2.45633602173877	-1.39777717764459	-0.00000161035208
C	4.92434088210641	-1.39792344434344	0.00002861306234
C	6.11673051662498	-0.70271187590053	0.00001928375403
C	6.11672646563517	0.70319359884309	0.00000045247791
C	4.92433367148532	1.39839725642730	-0.00000341584006
C	-0.00012357793349	-1.39632361564396	-0.00002427308300
C	-0.00013145831253	1.39674990770696	-0.00001516956508
H	2.45746707721085	2.48299465375046	-0.00003683564364
H	2.45749357334826	-2.48254422447513	0.00000564066058
H	4.92499686634016	-2.48156384529995	0.00004603202790
H	7.05581997465347	-1.24070522482940	0.00003300822268
H	7.05581109716230	1.24119536493984	-0.00000259199228
H	4.92496725405024	2.48203770720017	-0.00002001589536
C	-1.22932631368184	-0.72415490998369	-0.00001125106919
C	-2.45657145440812	-1.39781636125952	-0.00002188978188
C	-2.45658253438191	1.39822465416297	0.00000351190805
C	-4.92459612709671	-1.39796441137306	-0.00000496966634
C	-3.68473871530327	-0.72124592523394	0.00000164688684
C	-6.11699584793260	0.70314405525798	0.00001996391945
C	-3.68474310437036	0.72164992663081	0.00001504664013
C	-4.92460618380010	1.39836513849308	0.00002452804751
C	-1.22933368882898	0.72457373092169	0.00000038733524
C	-6.11698874908518	-0.70275743489119	0.00000198746436
H	-7.05607494356681	-1.24075824088944	0.00000595840869
H	-2.45770886926197	-2.48258589553619	-0.00003493432397
H	-4.92525566648091	-2.48160498179746	-0.00001615460573
H	-2.45772457463579	2.48299342105957	0.00000989955338
H	-4.92526929895790	2.48200988219130	0.00003374207890
H	-7.05608020392077	1.24114709856188	0.00002537045813
H	-0.00012154879787	-2.48129599389580	-0.00003631350483
H	-0.00013734331064	2.48172269546267	-0.00002300622294

6.2. 6,13-Diazapentacene (DAP)

6.2.1. Ground state equilibrium structures

Cartesian coordinates (in Å)

C	1.14647848902946	-0.72686199066283	-0.00013626784884
C	1.14647487071647	0.72755163586717	-0.00010853772341
C	2.37729198841024	1.41181673091718	-0.00006885391231
C	3.57837609996749	0.72698194522309	-0.00003248986194
C	3.57838194385535	-0.72631474605897	-0.00004065713384
C	2.37729866661794	-1.41113921281661	-0.00010466801889
C	4.83803155478640	-1.40662722973423	0.00003411087704
C	6.00687807698183	-0.71432504896207	0.00010625663829
C	6.00687445201499	0.71503295047236	0.00010840820421
C	4.83801301927063	1.40731702307262	0.00003837312127
N	0.00327745509242	-1.42055071175962	-0.00016401627186
N	0.00327082546912	1.42124411003437	-0.00009973985618
H	4.83679196063933	-2.49006638753776	0.00003869102838
H	6.95133764727945	-1.24315760732717	0.00017184198306
H	6.95132882057124	1.24387336000265	0.00016436906655
H	4.83674475124551	2.49075553884418	0.00004715012845
C	-1.13991981998461	-0.72685942264722	-0.00011592900244
C	-2.37073553461096	-1.41113665691046	-0.00006366633297
C	-2.37075789708789	1.41182235632077	-0.00005324649928
C	-4.83145698322910	-1.40665593392036	0.00007065422172
C	-3.57181811294502	-0.72631581976999	-0.00000272697640
C	-6.00033036293971	0.71497760911256	0.00007385718991
C	-3.57183223023040	0.72698457993166	-0.00001532516041
C	-4.83149239318609	1.40728499752270	0.00002253355504
C	-1.13993109999539	0.72755606538933	-0.00009442877790
C	-6.00031414444735	-0.71438320554793	0.00010653891725
H	-6.94476902137013	-1.24322437146682	0.00016536161779
H	-2.34660626704491	-2.49393063745784	-0.00005871463273
H	-4.83017494103305	-2.49009367921035	0.00009647077589
H	-2.34664353725921	2.49461581681444	-0.00004530048881
H	-4.83025077137238	2.49072262956464	0.00001046946286
H	-6.94479768040011	1.24379557387168	0.00010179841351
H	2.35318326364368	2.49460944366588	-0.00004737023491
H	2.35318007724474	-2.49393256373703	-0.00010494646808

6.2.2. Excited state (S_1) equilibrium structures

Cartesian coordinates (in Å)

C	1.14799054591157	-0.72407621929216	-0.00000858837851
C	1.14798099783167	0.72475196094601	-0.00000680364809
C	2.37671776795851	1.40074273806985	-0.00001888949156
C	3.60022993021883	0.72135741611464	-0.00000621922743
C	3.60023872587990	-0.72066636105099	0.00000088983262
C	2.37673268541727	-1.40006434413002	0.00000465888152
C	4.84074723153566	-1.39777659311467	0.00001097065302
C	6.03280285167920	-0.70277525132392	0.00001053740444
C	6.03279377157130	0.70349632093916	-0.00000022360766
C	4.84072824550851	1.39848541229489	-0.00001011744363
N	0.00328247911437	-1.42882680690424	-0.00000849836172
N	0.00327050183273	1.42948950073073	-0.00000399192828
H	4.84113549434528	-2.48115820632443	0.00002076438273
H	6.97174945435341	-1.24074452005647	0.00001775774743
H	6.97172910742907	1.24148476871625	-0.00000219541591
H	4.84109694495199	2.48186639321541	-0.00001952919620
C	-1.14143081669324	-0.72408659681945	-0.00000710030827
C	-2.37016979632521	-1.40007735370975	0.00000050046238
C	-2.37017408103182	1.40072602526538	0.00001732301213
C	-4.83418169336136	-1.39781126946218	-0.00000573472222
C	-3.59368105605965	-0.72068932267947	-0.00000128247102
C	-6.02624751655706	0.70344701332191	0.00000372100098
C	-3.59368298115104	0.72133339479799	0.00000656138540
C	-4.83419082724098	1.39844505270911	0.00001292109455
C	-1.14143529278132	0.72473949347373	-0.00000282097965
C	-6.02624376385014	-0.70282448992929	-0.00001039358312
H	-6.96518360188084	-1.24080536545868	-0.00001455383916
H	-2.34770499077484	-2.48348071314697	-0.00000290628993
H	-4.83455914126370	-2.48119163333000	-0.00001466785249
H	-2.34770810643468	2.48412850433953	0.00003170570133
H	-4.83457210770706	2.48182557436730	0.00002085405089
H	-6.96518964880140	1.24142461820572	0.00000371947243
H	2.35424477586441	2.48414525769082	-0.00002338644881
H	2.35426707621063	-2.48346725736571	0.00000501811183

6.2.3. Dimer ground state equilibrium structure

Cartesian coordinates (in Å)

C	5.71069673127335	1.99346769928280	0.28425638833355
C	5.70825520480356	0.55198754149671	0.26844261528450
N	4.57037841402807	-0.14008948544599	0.21288441656775
C	3.43400163223574	0.55754777309168	0.19561037875328
N	4.57453688691265	2.69033490427879	0.27462789879504
C	3.43685439977990	1.99795792855943	0.24320822350880
C	2.20046125681089	-0.12430282997272	0.12267620115178
C	1.01040980804511	0.56303684708932	0.11623442156455
C	2.20525351144739	2.68744224304493	0.25102684100751
C	1.01259590702015	2.00745313465850	0.18834333058023
C	-0.25589555075274	-0.11456425904192	0.03796111575243
C	-1.41555139396019	0.57594742211025	0.03732145349646
C	-1.41361387320743	2.00956428362585	0.11003402084230
C	-0.25191359672319	2.69370849177057	0.18223800259586
H	2.22559032553051	-1.20634223688130	0.07532164605994
H	2.23673499545074	3.76916385235314	0.30285578084533
H	-0.25340740367403	-1.19690849775980	-0.01926794584885
H	-2.36090518741040	0.05038254672665	-0.02395691349240
H	-2.35705023132252	2.54221329764161	0.10155172865012
H	-0.24793484374247	3.77670574019218	0.23033549285417
C	6.94427035959474	2.67856966523501	0.31188918030093
C	8.13427016188061	1.99221784996777	0.35621272119622
C	8.13043785166494	0.54568861197504	0.37251140462462
C	6.93939544633157	-0.13676644952694	0.31617200170170
H	6.91869220977705	3.76134374567469	0.30134869444877
C	9.40050738729695	2.67323520567038	0.38590330512404
H	6.90976293734391	-1.21949089492819	0.31220676200050
C	10.55882199460355	1.98303393569243	0.44584943589475
H	9.40113405900379	3.75710783623212	0.37030642793865
C	10.55533640622627	0.54816121191611	0.48305415687061
H	11.50386290845158	2.51141130185387	0.47540352270760
C	9.39367546024453	-0.13903249996840	0.44592580138071
H	11.49823681432675	0.01718243944086	0.53902331259520
H	9.38855502373200	-1.22275194158029	0.46844057555433
C	-0.07702107251848	-0.81978814816405	3.58504911940739
C	4.12169648076848	-0.84850015778820	3.59810243683896
C	8.18802530311906	3.96641870795795	3.85946975177951
C	6.81423192034997	3.96101454412228	3.80657918249248
C	2.74742863882520	-0.85307802182458	3.58110146629128
C	0.58375984338915	0.35651899088251	3.62123082724130
C	2.71097682252880	1.58628515102551	3.65995731847453
C	0.64143283356310	-2.06116186832324	3.53280746600170
C	2.02196124155230	0.39788250264754	3.61911210262141
C	1.99208170413142	-2.07699874625413	3.52946742108601

C	8.91241214521837	2.71452348150355	3.87511646195470
C	8.22523068923608	1.52617643063602	3.81365324809156
C	10.34895572712357	2.75695083100252	3.95272004519035
C	6.09182450110658	2.74830830828612	3.75849954398472
C	6.81449468179453	1.50144014348547	3.74877367771422
C	11.00940752640311	3.93325302767272	3.99677768846509
C	4.12296954690116	1.61022139825990	3.67995216338847
C	4.84495518201015	0.36298772892775	3.65415566776611
C	8.94231197228537	5.19153354775428	3.90936736147353
C	10.29129764186994	5.17597443846290	3.96971350468033
N	6.17776010574025	0.33151094999296	3.68857886345120
N	4.76012490935616	2.77988188131581	3.72851265432614
H	2.18928413841055	2.53518932018867	3.68729670509235
H	8.39753867011377	6.12863756488344	3.90042858013501
H	6.24577016394490	4.88401791118121	3.80721946560308
H	10.84737081590216	6.10517966409490	4.01072930349765
H	10.88750472577217	1.81597353009986	3.97550570133470
H	12.09077796575029	3.95048504184235	4.06197797981745
H	8.74760934674491	0.57700496841107	3.81331605190464
H	4.68830914488904	-1.77192439610731	3.57228584437223
H	0.08380399223415	-2.98981671582299	3.49901285714137
H	2.53696372866673	-3.01329405701387	3.49374109843149
H	-1.16005912707004	-0.83664150902840	3.58753223926986
H	0.04295183806052	1.29628208831340	3.64630380103688
